# Periconceptional Heat Stress of Holstein Dams Is Associated with Differences in Daughter Milk Production during Their First Lactation

**DOI:** 10.1371/journal.pone.0148234

**Published:** 2016-02-03

**Authors:** Britni M. Brown, Jon W. Stallings, John S. Clay, Michelle L. Rhoads

**Affiliations:** 1 Department of Animal and Poultry Sciences, Virginia Polytechnic Institute and State University, Blacksburg, Virginia, United States of America; 2 Department of Statistics, North Carolina State University, Raleigh, North Carolina, United States of America; 3 Department of Animal Science, North Carolina State University, Raleigh, North Carolina, United States of America; University of British Columbia, CANADA

## Abstract

The fertility of lactating Holstein cows is severely reduced during periods of heat stress. Despite this reduction in fertility, however, some inseminations conducted during heat stress result in successful pregnancies from which heifer calves are born. Many of these heifer calves are retained and raised to enter the milking herd as replacement animals. Heat stress experienced by these females around the time they were conceived may confer long-lasting effects that alter subsequent milk production capacity. The objective of this study was to examine the relationship between periconceptional heat stress and subsequent milk production of primiparous cows. National Dairy Herd Improvement Association data was obtained from Dairy Records Management Systems. Records included Holstein cows that had completed at least one lactation in one of three states with large populations of dairy cattle and which are known for having hot, humid summers: Georgia, Florida or Texas. Dates of conception were calculated by subtracting 276 d from the recorded birth date of each individual cow. Records for cows conceived within the months of June, July, and August were retained as heat stress-conceived (HSC) cows (n = 94,440); cows conceived within the months of December, January, and February were retained as thermoneutral-conceived (TNC) contemporaries (n = 141,365). In order to account for the effects of environmental conditions on total milk production for a given lactation, cows were blocked by season of calving (winter, spring, summer or fall). Adjusted 305-day mature-equivalent milk production was evaluated with a mixed model ANOVA using SAS, in which random effects were used to account for variability between herds. Of the cows that calved in the summer, fall and winter, TNC cows had higher milk yield than the HSC cows in all states. Interestingly, the cows that calved in the spring presented a unique relationship, with HSC cows producing more milk. Overall however, heat stress at the time of conception is associated with lower milk production during the first lactation. While this association does not prove cause and effect, it does provide justification for additional investigation into whether heat stress around the time of conception results in long-term, detrimental consequences for the conceptus.

## Introduction

Dairy cattle have historically been selected for traits that contribute to productivity. In general, body mass has increased to accommodate a large mammary system and other internal organs that contribute to milk synthesis. Unfortunately, this selection strategy has theoretically decreased heat tolerance of dairy cattle because the heat produced to meet maintenance needs is directly proportional to the body weight and surface area of an animal [1]. Therefore, as a cow increases in size, metabolic heat production increases. Metabolic heat production also escalates as the productive capacity of a dairy cow increases. Further compounding the issue, most of the selection for dairy production takes place in temperate climates [2] and does not account for individual performance in various environmental conditions.

In the absence of selection for heat tolerance, modern dairy cattle remain highly susceptible to the effects of heat stress. In the United States alone, approximately $1 billion is lost annually as a result of poor performance during periods of heat stress [3]. Poor performance encompasses a myriad of heat stress-related consequences, including depressed feed intake, increased ailments, reduced milk production and decreased fertility [2,4,5]. One of the primary focuses of heat stress-related research has been to improve fertility during periods of heat stress. In sheep, however, periconceptional stress in the form of under- or over-nutrition has been linked with postnatal alterations in physiology and behavior [6,7]; some of which have been shown to persist into adulthood. If heat stress at the time of conception in dairy cattle causes comparable alterations, lifetime productivity and profitability could be affected. This was indeed observed in a previous study where the milk production records of multiparous dairy cows were examined across three lactations [8]. The multiparous population was chosen for that analysis because cows that have remained in a herd for at least three lactations have been subjected to substantial selection and culling and because multiple lactations equates with multiple opportunities to affect herd productivity. In almost all cases, multiparous dairy cows that had been conceived during summer months produced less milk than their counterparts that were conceived in the winter months, although the differences in milk production were relatively modest. Since the selection criteria of the previous study required that all cows complete at least three lactations, the cows retained in the analysis were superior to their counterparts that were culled prior to completing three lactations. Thus, it is unclear whether the association between heat stress at the time of conception and subsequent milk production is similar or maybe even amplified in a population of cows that have not yet been subjected to heavy selection and culling pressure.

Therefore, the objective of this study was to evaluate the effects of periconceptional heat stress on first lactation milk yield. We hypothesized that females that had experienced heat stress around the time of their own conception would exhibit altered milk production, and that these alterations (i.e., increase or decrease) may be dependent upon the season in which the cow reaches peak milk production. For this particular study we chose to focus on primiparous cows because, for the most part, the first lactation population within a herd has not yet been subjected to selection via culling. This is an important distinction because it allows evaluation of a representative sample of all cows rather than just the high performing cows that are retained for multiple years.

## Materials and Methods

Dairy Herd Improvement Association data was received from Dairy Records Management Systems (Raleigh, NC) for dairy cows in three states known to have hot and humid summers: Georgia, Florida and Texas. Conception date for each individual cow was calculated assuming that the average gestation length of a Holstein is 276 d. [9]. The month in which conception took place was used as an indicator of heat stress at the time of conception. Cows that were conceived during December, January and February were considered thermoneutral conceived (TNC), while cows conceived during June, July and August were considered heat stress conceived (HSC). Since fall (September, October and November) and spring (March, April and May) weather conditions are intermediate and highly variable, cows conceived during these months were excluded from analysis.

Thermoneutral and heat stress conditions for the TNC and HSC cows were verified by calculating the temperature-humidity index (THI) for each season within each state and was described previously [8]. Climatic data was received from the National Climatic Data Center (http://www.ncdc.noaa.gov/), and included hourly temperature, humidity and dew-point observations from 1999 through 2011 for locations across each state (Table 1). Temperature-humidity index values were calculated using an equation derived for use with dairy cattle [10].

$$THI=T_{\text{dry bulb}}+\left(\left(0.36*T_{\text{dew point}}\right)+41.2\right)$$

**Table 1:** Weather records were collected from specific weather stations in Georgia, Florida and Texas and used to calculate mean temperature-humidity index (THI) values for the summer and winter in each state.

State	Weather Station Name	Latitude/Longitude
Georgia	Atlanta Hartsfield International Airport	33.63010°, -84.44180°
	Macon Middle GA Regional Airport	32.68470°, -83.65270°
Florida	Tallahassee Regional Airport	30.39306°, -84.35333°
	West Palm Beach International Airport	26.68470°, -80.09940°
Texas	Fort Worth Naval Air Station	32.76667°, -97.45000°
	San Antonio International Airport	29.54430°, -98.48390°
	Lubbock International Airport	33.66560°, -101.82310°
	Midland International Airport	31.9475°, -102.2086°

A THI value was calculated for each hourly observation, which was then used to determine the mean THI for each season.

For an appropriate analysis of milk production, we not only considered the season in which the cow herself had been conceived, but also the season in which she calved. Season of calving (SOC) was determined using the month of each calving date. The seasons were designated as spring: March, April, May; summer: June, July, August; fall: September, October, November; and winter: December, January and February.

Statistical analyses were performed using SAS (SAS Institute Inc, Cary, NC). Due to the large size of the data sets, each state was analyzed separately, and was restricted to only include cattle that had completed at least one lactation, with 305-day adjusted mature-equivalent milk between 2,268 and 20,412 kg within herds containing at least 10 cows that fit the selection criteria. For each state, the total numbers of cows included in the analysis are listed in Table 2. Frequency distributions of mature-equivalent milk by heat stress category and season of calving for Georgia cattle are shown in the Supporting Information (S1 Fig). Cattle in Florida and Texas had comparable distributions. The distributions were slightly right-skewed due to cattle with high mature-equivalent milk, although such cases were very rare relative to the majority of the population.

**Table 2:** Number of cows from Georgia, Florida and Texas that were included in the analyses of thermoneutral-conceived (TNC) and heat stress-conceived (HSC) Holstein cattle.

	Georgia	Florida	Texas	Total
TNC cows	36,394	44,551	60,420	141,365
HSC cows	22,869	24,914	46,657	94,440
Total	59,263	69,465	107,077	235,805

The analysis focused on estimating the effects of heat stress, season of calving (SOC), and their interaction on milk production (305-day adjusted mature-equivalent milk). For a given state, let Y_ijkl_ denote the milk production of the *l*-th cow in herd *i* and heat stress category *j* (HSC or TNC) having calved during season *k*. The indices *i* and *l* depend on the state and herd analyzed, while *j* = 1 or 2 and *k* = 1, 2, 3, or 4, corresponding to the two treatment categories and four seasons. The linear mixed model for response Y_ijkl_ was:

$$Y_{ijkl\;=}\mu \;+\;H_{i}+\;\tau_{j}+\;\theta_{k}+\;{\left(\tau \theta \right)}_{jk}+\;e_{ijkl}$$

where μ is the overall intercept; H_i_ is the effect due to different herds; τ_j_ is the effect due to the HSCONCP category; θ_k_ is the effect due to SOC; (τθ)_jk_ is the interaction effect between HSCONCP and SOC; and e_ijkl_ is the usual residual error term. In order to generalize our conclusions, and reflect the fact that milk production from cows in the same herd is likely correlated, we assumed H_i_ was a random effect generated from a normal distribution with mean 0 and variance σ_h_^2^.

SAS/STAT^®^ 9.4 software’s PROC MIXED was used to perform the appropriate tests for the effects of heat stress and SOC using the REML (residual maximum likelihood) estimation procedure. Plots of the studentized residuals were produced to check the validity of the models and the statistical tests, and can be found in the Supporting Information (S2 Fig). Contrasts comparing HSC and TNC at different SOC levels were then performed to investigate the interaction between heat stress and SOC. Due to the large sample size, results were considered significant with a P-value <0.01, and a tendency for significance was declared at P<0.05.

## Results and Discussion

Lactating dairy cattle are extremely susceptible to heat stress. Traditionally, a THI value of 72 has been used as a threshold to predict whether or not dairy cattle are experiencing heat stress; meaning that at a THI of 72 or above, producers should expect a decrease in milk yield. However, this threshold was based upon a retrospective analysis of studies conducted in the 1950’s and 1960’s. A recent re-evaluation of the THI indicates that high-producing dairy cattle are much more sensitive to environmental conditions than previously thought. Milk production actually begins to decline when the minimum THI does not fall below 65, or the average THI is 68 or greater [11]. The average seasonal THI values were calculated for each state used in this study, and were previously reported [8]. Regardless of which threshold is used as a designation of heat stress (THI of 72 or 68), it was clear that, overall, summer conditions exceeded the threshold while winter conditions did not.

The milk production variable that was selected for analysis in this study was the 305-day adjusted mature-equivalent milk. This variable was chosen, because amongst other factors, this calculation includes correction factors for the age of the animal at the time of calving. The age of the animal at the time of calving was a concern because it was a potential confounding factor that is inherently imbedded in the data. For example, the majority of the HSC cows that calved in the summer would have been approximately 24 months of age at calving while HSC cows that calved in the spring would have either been 21 months of age or 33 months of age. It is important to recognize, however, that even if this correction were not available, the results of this study would remain relevant because this study is representative of on-farm scenarios.

The relationship between heat stress at the time of conception and subsequent milk production is complex. Inherently, there are many factors that are difficult to account for when analyzing production records across numerous herds. Fortunately, since much of the variation in management and nutrition is herd specific, these factors were accounted for in the statistical analyses. Differences in milk production between TNC cows and HSC cows were observed across states and seasons of calving, and the directionality of these differences (favoring TNC or HSC cows) were remarkably consistent across states. This degree of consistency suggests that comparable responses could be expected anywhere world-wide where similar conditions exist.

For all three states, there was a significant interaction between heat stress at the time of conception and SOC (P<0.001 for each Georgia, Florida and Texas). Estimates of the herd-to-herd variation were all found to be significant, accounting for between 23% (Florida) to 38% (Texas) of the total variation. Normal QQ-plots of the residuals (see Supporting Information) showed slight deviations from normality, but do not appear to compromise the validity of tests and confidence intervals, especially considering the large sample size.

The TNC cows produced significantly more milk than HSC cows in most instances. When considering cows that calved in the summer, fall and winter, those that were TNC produced between 172 ± 43 and 423 ± 39 kg more milk than their HSC counterparts over the course of their first lactation (P<0.001). This relationship was significant in all three states (Table 3). Although the magnitude of the differences varies from state to state and season to season, the fact that TNC cows consistently produced more than the HSC cows during the summer, fall and winter is notable, and worthy of consideration. Milk production of the spring-calving cows was also affected by season of conception, but in this instance, HSC cows produced more milk than those that were TNC (P<0.001, P<0.001 and P<0.05 for Georgia, Florida and Texas, respectively; Table 3).

**Table 3:** Differences in mature-equivalent milk yield between thermoneutral conceived (TNC) and heat stress conceived (HSC) cows in Georgia, Florida and Texas.

State	Season of Calving	P-Value	HSC Mature-Equivalent Milk Produced (kg)	Difference in Mature-Equivalent Milk Produced^1^ (kg)	SE^2^	99% Lower Limit	99% Upper Limit
Georgia	Spring	<0.001	9306	-131	40	-234	-29
	Summer	<0.001	9414	172	43	61	282
	Fall	<0.001	9339	384	38	287	481
	Winter	<0.001	9120	176	47	54	298
Florida	Spring	<0.001	8985	-182	42	-291	-73
	Summer	<0.001	9055	415	44	300	530
	Fall	<0.001	9033	423	39	321	524
	Winter	<0.001	8796	197	48	73	321
Texas	Spring	0.488	9163	-20	29	-95	54
	Summer	<0.001	9224	241	36	148	334
	Fall	<0.001	9108	391	27	321	462
	Winter	<0.001	8904	224	38	127	320

^1^Positive values indicate the superior performance of TNC cows, while negative values indicate the superior performance of HSC cows.

^2^SE = standard error.

Unexpectedly, spring-calving cows that were HSC not only met, but actually exceeded the milk production of their TNC counterparts. The cows that calved during the spring would have traversed the transition period during mild environmental conditions and subsequently achieved peak milk production during the late spring or summer heat stress. Under these conditions, the HSC cows outperformed the TNC cows. This suggests that heat stress around the time of conception may improve thermotolerance and, thereby, offer an advantage to the HSC cows during subsequent periods of heat stress. Potential mechanisms responsible for this advantage in milk production are currently unknown, but are likely related to the ability to thermoregulate. For example, pigs whose dams were exposed to heat stress during gestation have a lower skin to BW ratio. This reduction is thought to improve their ability to dissipate heat [12]. Similar adaptations likely occur in cattle and would result in greater thermotolerance during adulthood. If true, this would mean that even though cattle conceived during heat stress produce less milk in most instances, they have an adaptive advantage when exposed to heat stress. These potential adaptations allow them to better maintain milk production during heat stress. Lower milk production capacity of the HSC cows would also reduce metabolic heat production, and could contribute to greater thermotolerance. This putative thermotolerance would be most important for cattle whose peak in milk production occurs during heat stress.

The differences in milk production (kg of milk) between the TNC and HSC cattle in the current study is greater than those reported in a previous study of lactation records from cows that had completed at least three lactations. Thermoneutral conceived cows from those previously reported analyses produced between 82 ± 42 and 399 ± 61 kg more milk than their HSC counterparts [8]. Furthermore, the spring-calving cows from the previous analyses did not exhibit the same unique relationship observed here. Across all lactations and locations, there was only one instance where the HSC cows merely tended to produce more milk than the TNC cattle (first lactation spring-calving cows in Florida; [8]). The discrepancies in production advantage are most certainly due to the differences in the inclusion criteria between studies. In the previous study, only cows that completed three or more lactations were eligible for inclusion in the analyses. Over the course of those three lactations, the population of cattle would have been subjected to immense selection and culling so that only the best cows remain at the end of three lactations. The population included in the current analysis was much more inclusive because cows needed only complete their first lactation, and therefore would have undergone only minimal selection and culling for milk production. The fact that the production difference between TNC and HSC cattle was of a greater magnitude in this analysis of primiparous cows (a primarily unselected population), and the spring-calving advantage differed between studies suggests that fewer HSC cows are retained within the herd for three or more lactations. Additional research is needed to confirm this assertion.

The paternal contribution to relative thermotolerance of the HSC cows was not evaluated in this experiment. Since more than 235,000 records from three states over 10 years were retrieved for this experiment, a wide variety of paternal genetic/epigenetic profiles would have been represented in the daughters retained in the analysis (including those sired by herd bulls). Recent experiments have shown that within the Holstein breed, certain genetic profiles are indeed related to thermotolerance [13,14]. However, within the breed, these genetic profiles are not necessarily related to an increased likelihood to produce a pregnancy during heat stress, and therefore, should have no effect on the distribution of sires across seasons in the current analysis. A sire effect on pregnancy rate during heat stress has been detected when Holstein cows are inseminated with semen from Bos indicus bulls [15]. Even in such extreme crossbreeding systems, however, the improvement in pregnancy rate is relatively modest. In fact, recent research has shown that the breed origin of the oocyte, rather than that of the sperm, has the greatest impact on embryo survival during heat stress [16]. Therefore, it is unlikely that the paternal genetic and/or epigenetic contribution differed between the TNC and HSC cows in this study.

The results of this experiment demonstrate an association between periconceptional heat stress and subsequent production of primiparous Holstein cows. This information provides a valuable basis for future studies investigating the nature of this relationship. If periconceptional heat stress is indeed a direct cause of limited milk production and is related to a higher rate of culling as suggested here, this consideration will need to be incorporated into on-farm breeding and management decisions. Biological mechanisms responsible for such a relationship could be many-fold but would likely include an epigenetic component. Regardless of the mechanism(s), however, characterization of this relationship will provide important insight into the non-genomic factors affecting the long-term productivity of dairy cattle.

## Supporting Information

S1 Fig Frequency histogram of mature-equivalent milk produced by Georgia cattle by season of calving and heat stress category. (TIF)

S2 Fig Normal QQ-plots of studentized residuals for linear mixed models by state. (TIF)
