# Supplementary figures and images for: Periconceptional Heat Stress of Holstein Dams Is Associated with Differences in Daughter Milk Production during Their First Lactation
Source: PLoS One. 2016 Feb 3;11(2):e0148234. doi: 10.1371/journal.pone.0148234 (PMC4739617; doi:10.1371/journal.pone.0148234)

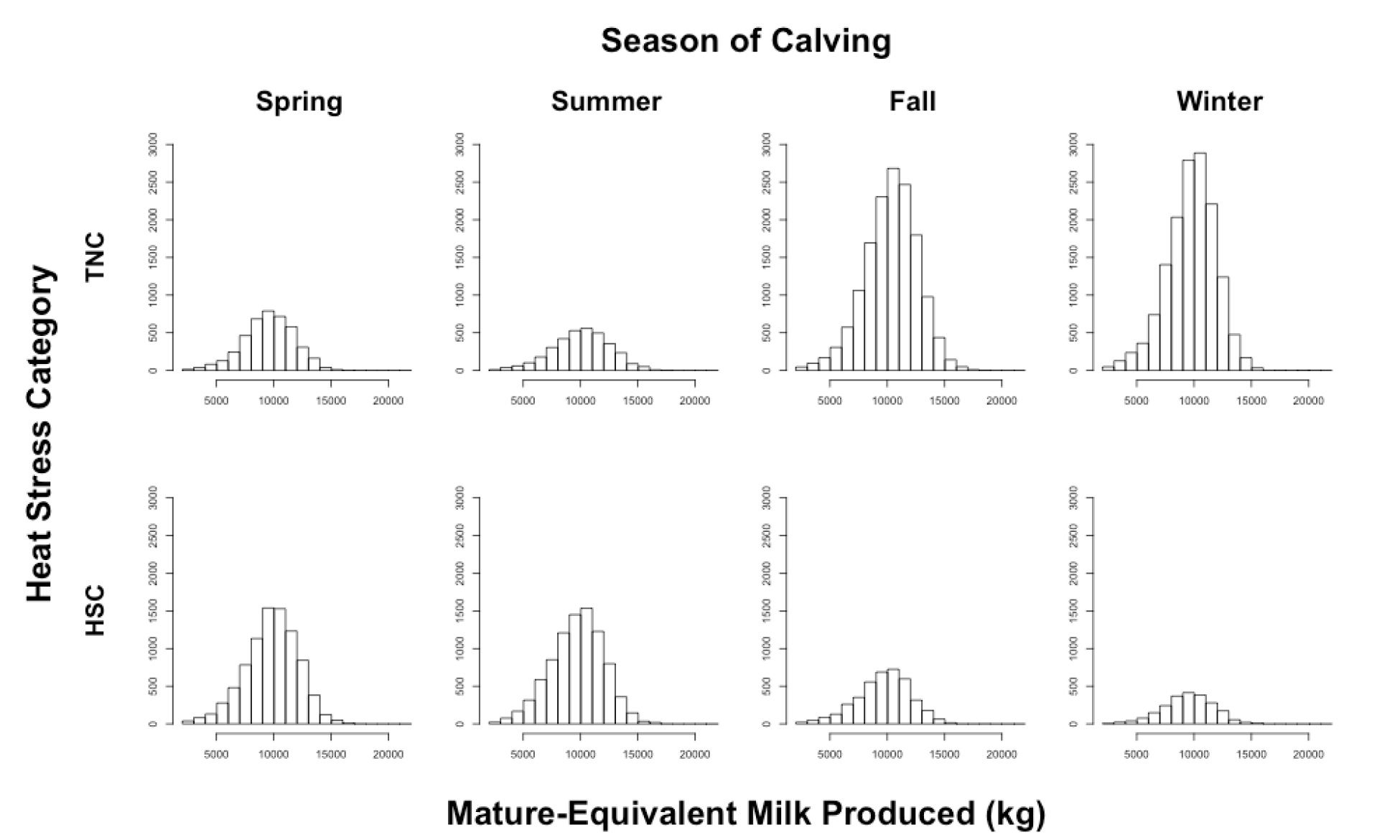

Supplement: S1 Fig — (TIF) [file pone.0148234.s001.tif]

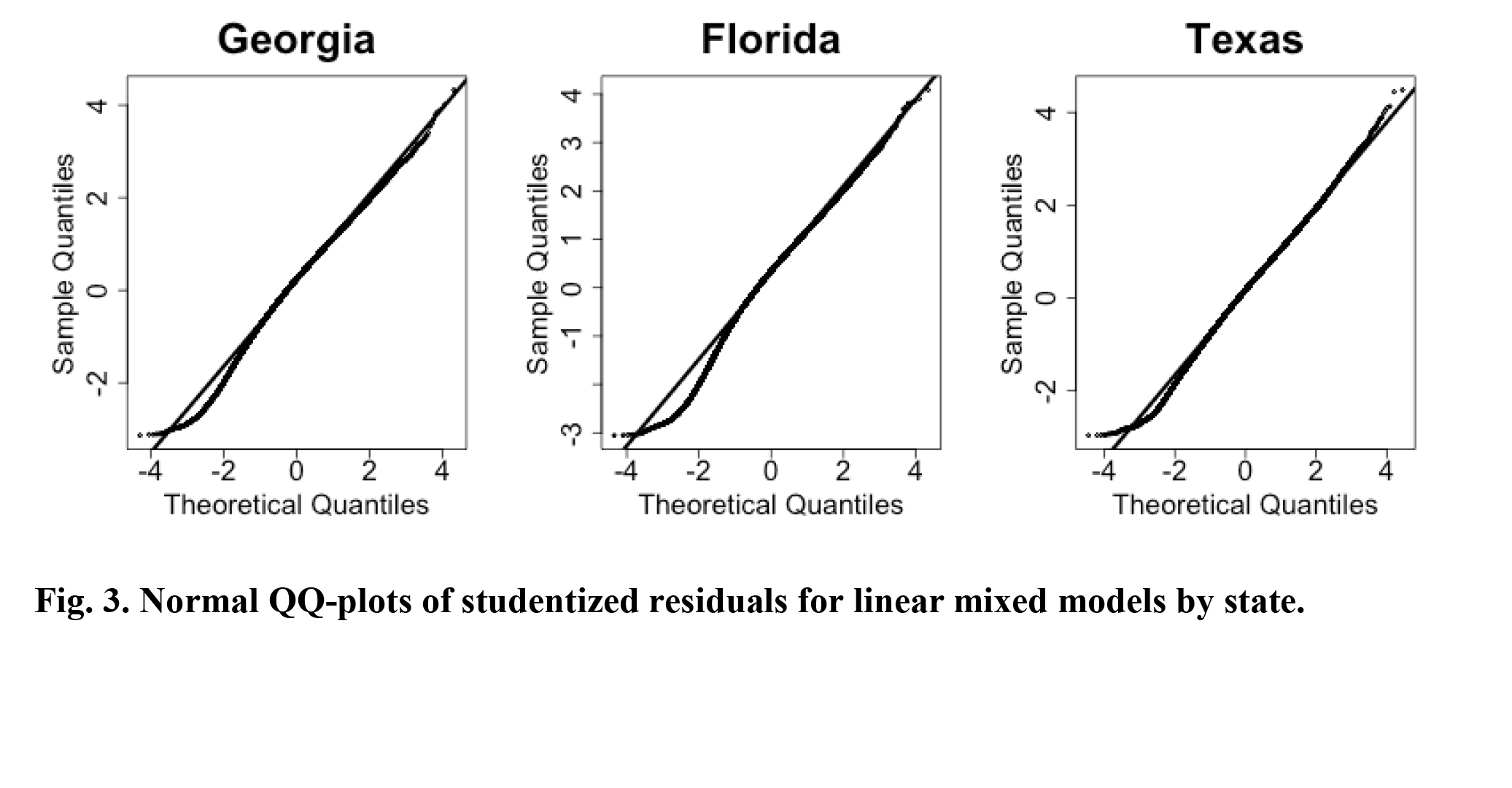

Supplement: S2 Fig — (TIF) [file pone.0148234.s002.tif]
